# Detection of the first G6P[14] human rotavirus strain in an infant with diarrhoea in Ghana

**DOI:** 10.1186/s12985-016-0643-y

**Published:** 2016-11-10

**Authors:** Susan Damanka, Belinda Lartey, Chantal Agbemabiese, Francis E. Dennis, Theophilus Adiku, Kofi Nyarko, Michael Ofori, George E. Armah

**Affiliations:** 1 Department of Electron Microscopy and Histopathology, Noguchi Memorial Institute for Medical Research, College of Health Sciences, University of Ghana, Legon, Accra Ghana; 2 Department of Microbiology, School of Biomedical and Allied Health Sciences, College of Health Sciences, University of Ghana, Accra, Ghana; 3 Department of Epidemiology and Disease Control, School of Public Health, College of Health Sciences, University of Ghana, Legon, Accra Ghana; 4 Department of Immunology, Noguchi Memorial Institute for Medical Research, College of Health Sciences, University of Ghana, Legon, Accra Ghana

**Keywords:** Rotavirus, Gastroenteritis, Genotyping, Ghana

## Abstract

**Background:**

Rotaviruses with G6P[14] specificity are mostly isolated in cattle and have been established as a rare cause of gastroenteritis in humans. This study reports the first detection of G6P[14] rotavirus strain in Ghana from the stool of an infant during a hospital-based rotavirus surveillance study in 2010.

**Methods:**

Viral RNA was extracted and rotavirus VP7 and VP4 genes amplified by one step RT-PCR using gene-specific primers. The DNA was purified, sequenced and genotypes determined using BLAST and RotaC v2.0. Phylogenetic tree was constructed using maximum likelihood method in MEGA v6.06 software and statistically supported by bootstrapping with 1000 replicates. Phylogenetic distances were calculated using the Kimura-2 parameter model.

**Results:**

The study strain, GHA-M0084/2010 was characterised as G6P[14]. The VP7 gene of the Ghanaian strain clustered in G6 lineage-III together with artiodactyl and human rotavirus (HRV) strains. It exhibited the highest nucleotide (88.1 %) and amino acid (86.9 %) sequence identity with Belgian HRV strain, B10925. The VP8* fragment of the VP4 gene was closely related to HRV strains detected in France, Italy, Spain and Belgium. It exhibited the strongest nucleotide sequence identity (87.9 %) with HRV strains, PA169 and PR/1300 (Italy) and the strongest amino acid sequence identity (89.3 %) with HRV strain R2775/FRA/07 (France).

**Conclusion:**

The study reports the first detection of G6P[14] HRV strain in an infant in Ghana. The detection of G6P[14], an unusual strain pre-vaccine introduction in Ghana, suggests a potential compromise of vaccine effectiveness and indicates the necessity for continuous surveillance in the post vaccine era.

**Electronic supplementary material:**

The online version of this article (doi:10.1186/s12985-016-0643-y) contains supplementary material, which is available to authorized users.

## Background

Group A rotaviruses (RVAs) are the leading cause of severe, dehydrating, acute diarrhoea in children <5 years of age worldwide and are estimated to be responsible for 453,000 deaths annually, with a disproportionate number of these deaths occurring in sub-Saharan Africa and South East Asia [1]. The World Health organization (WHO) recommended global use of rotavirus vaccines as one of the effective interventions to reduce RVA-associated diseases [2]. Currently, two RVA vaccines, RotaTeq (Merck & Co., Inc., United States) and Rotarix (GlaxoSmithKline Biologicals, Belgium) have been licensed and introduced into routine childhood immunisation programmes.

Rotavirus, a genus of the *Reoviridae *family, contains a genome of 11 segments of double-stranded RNA (dsRNA) and are classified in a binary system based upon the main neutralization antigens, namely, the spike protein (VP4) and the major outer capsid glycoprotein (VP7) [3]. VP7 and VP4 nucleotide sequences define G and P genotypes, respectively, and carry independent neutralization-specific epitopes [3]. Recently, the Rotavirus Classification Working Group (RCWG) proposed an extensive RVA classification system that takes into account all 11 rotavirus genome segments [4]. Prior to the present study, at least 27 G genotypes and 37 P genotypes have been identified globally and approximately 73 G/P genotype combinations of rotaviruses have been reported to infect humans [5]. Among these, only six G/P-genotype combinations; G1P[8], G2P[4], G3P[8], G4P[8], G9P[8] and G12P[8] account for most of the HRV strains detected globally [6]. However, several epidemiological studies have reported the sporadic detection of unusual rotavirus G-genotypes (G5-G6, G8, G10, G11 and G20) and P-genotypes (P[1]-P[3], P[5], P[7], P[9]-P[11], P[14], P[19] and P[25]) in humans [3, 6, 7]. Many of these unusual rotavirus genotypes believed to be of animal origin have been introduced into the human population through interspecies transmission and/or reassortment events [3, 8].

Rotaviruses with G6 specificity are recognised as common serotype in cattle and other ruminants and have been found to be an infrequent cause of human disease. Bovine-like human G6 rotavirus strains PA151 and PA169 were first identified in 1987 and 1988 respectively from two Italian children hospitalised with acute gastroenteritis [9]. These two strains were characterised as G6P[9] (strain PA151) and G6P[14] (strain PA169). Subsequently, an increasing number of G6 HRV strains have been detected in combination with other P genotypes across the globe (Hungary, Australia, United States and India) [10–13]. G6 HRV strains are reported to be found mainly in combination with P[14] rotaviruses (commonly detected in antelope, cattle, rabbits, goats, sheep and guanacos) [14]. Previous reports in Africa, documented the isolation of G6 rotavirus strains only in animals [15]. The first detection of G6P[14] HRV strain in Africa was in 2004 from the stool of a child from Egypt [7].

In our previous study, rotavirus was detected in the stool of a four month old child hospitalised with severe, acute dehydrating gastroenteritis at Maamobi Polyclinic in Accra, Ghana in August 2010. This sample exhibited the classical human rotavirus group A migration pattern (4-2-3-2) with a long electrophoretype by Polyacrylamide Gel Electrophoresis (PAGE) (data not shown). However, the amplified VP7 and VP8* fragment of the VP4 gene could not be genotyped using the routine HRV genotype specific primers. In this study, the G and P genotypes of strain GHA-M0084/2010 were identified by sequencing.

## Results

**Fig. 1:**
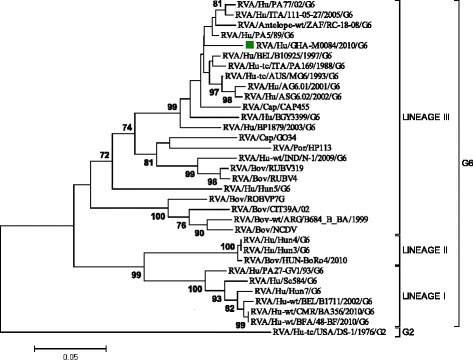
Phylogenetic tree constructed from nucleotide sequence of Ghanaian G6 strain GHA-M0084/2010 and reference G6 strains by the maximum likelihood method using MEGA 6.06 software and rooted with the G2 human rotavirus strain, DS-1. Variation scale (nucleotide substitution per site) is indicated below the phylogenetic tree. Percentage bootstrap support is indicated by values at each node, and values ≥70 % are shown. Study strain GHA-M0084/2010 is indicated by a filled *green square*. Reference sequences used in the analysis were obtained from the GenBank database. Phylogenetic distance was measured by Kimura two-parameter model. Phylogenetic tree was supported statistically by bootstrapping with 1000 replicates

The study strain GHA-M0084/2010 was characterised as G6P[14]. Phylogenetic analysis showed the VP7 gene of the Ghanaian G6P[14] strain clustered in lineage III together with randomly selected artiodactyl and human G6 strains (Fig. 1). It clustered in G6 lineage-III together with HRV strains isolated in Italy (RVA/Hu/PA5/89, RVA/Hu-tc/ITA/PA169/1988), Belgium (RVA/Hu/BEL/B10925/1997), and with artiodactyl strain, (RVA/Caprine/CAP455) isolated in South Africa (Fig. 1). The VP7 gene of the study strain shared the strongest nucleotide (88.1 %) and amino acid (86.9 %) sequence identity with the Belgian HRV strain, B10925 detected in 1997. However, it shared very low nucleotide (74.9 %) and amino acid (74.9 %) sequence identity with West African isolates BA356 (Cameroon) and 48-BF (Burkina Faso) (Table 1).

**Table 1:** Comparison of nucleotide and amino acid sequence identity of Ghanaian G6 and published G6 strains

Nucleotide identity (%)																				
**RV STRAIN**	G-TYPE	PA169	AG6	ASG6	MG6	PA5	B10925	BA356	48-BF	PA77	N-1	Hun5	Hun4	ROBVP7G	NCDV	Hun7	BoRo4	PA27	Hun3	**GHA-M0084**
PA169	G6		94.9	96.5	97.5	97.2	97.8	79.2	78.8	96.1	85.1	86.2	79.8	82.5	82	79.5	80.1	79.9	79.8	87.1
AG6	G6	96.7		97.2	97.1	94.1	94.5	76.9	76.5	93.3	83.9	85.2	78.4	81.4	81	77.2	78.8	78	78.4	85
ASG6	G6	99.1	96.7		98.7	96	96.1	78.6	78.2	95.2	85.8	86.5	79.5	82.6	82.4	78.4	79.9	79.4	79.5	86.2
MG6	G6	99.5	97.1	99.5		96.7	97.1	78.8	78.4	95.9	86.3	86.5	79.8	83.2	82.5	79.1	80.2	79.9	79.8	87.1
PA5	G6	98.7	96.3	98.7	99.1		97.1	79.8	79.4	98	85.4	86.3	79.9	83.2	82.5	79.9	80.2	80.2	79.9	87.7
B10925	G6	98.7	96.3	98.7	99.1	99.1		78.8	78.4	96.5	86.3	86.3	79.5	83.3	82.6	79	79.7	79.5	79.5	88.1
BA356	G6	92.2	89.3	92.6	92.2	91.4	91.4		99.5	79.7	78.8	78.6	86.2	79.8	80.2	96	85.9	93.4	86.2	74.9
48-BF	G6	92.2	89.3	92.6	92.2	91.4	91.4	100		79.2	78.4	78.7	86.3	79.7	80.1	96.1	85.8	93.5	86.3	74.9
PA77	G6	99.1	96.7	99.1	99.5	99.5	99.5	91.8	91.8		85.4	86.5	79.9	83.1	82.8	79.8	80.1	80.1	79.9	87.6
N-1	G6	96.7	94.2	96.7	97.1	96.7	96.7	92.6	92.6	97.1		84.4	80.1	83.6	82.6	79.9	79.8	79.9	80.1	79.2
Hun5	G6	98.3	95.9	98.3	98.7	98.3	98.3	91.8	91.8	98.7	97.1		81.7	81.8	80.2	79.7	81.3	79.9	81.7	78.7
Hun4	G6	92.6	89.7	92.6	92.6	91.8	92.2	93.4	93.4	92.2	92.2	92.2		81	80.5	86.1	98.6	88.2	100	75.2
ROBVP7G	G6	93	90.6	92.2	92.6	92.6	92.6	89.3	89.3	93	92.2	91.8	89.7		93.7	79.9	80.6	80.3	81	76.5
NCDV	G6	91.8	89.3	91	91.4	91.4	91.4	88.9	88.9	91.8	91	90.6	89.3	95.9		80.3	80.2	80.7	80.5	75.8
Hun7	G6	93	90.2	92.6	93	92.2	92.2	98.7	98.7	92.6	93.4	92.6	93.8	90.2	89.7		85.4	95	86.1	75
BoRo4	G6	92.6	89.7	92.2	92.6	91.8	92.2	93	93	92.2	92.2	92.2	99.1	89.7	89.3	93.8		88.2	98.6	75
PA27	G6	92.6	89.7	92.2	92.6	91.8	91.8	97.1	97.1	92.2	93.4	92.2	94.2	90.6	90.2	97.9	94.2		88.2	75.2
Hun3	G6	92.6	89.7	92.6	92.6	91.8	92.2	93.4	93.4	92.2	92.2	92.2	100	89.7	89.3	93.8	99.1	94.2		75.2
**GHA-M0084**	G6	85.7	83.2	85.7	86.1	86.1	86.9	80.4	80.4	86.5	85.7	85.7	81.2	81.2	80	81.2	81.2	80.8	81.2	
**Amino acid identity (%)**																				

Comparison of the deduced nucleotide (right, upper) and amino acid (left, below) identity of the VP7 gene of the Ghanaian G6 rotavirus strain with G6 reference strains from the GenBank. Study isolate is boldface type

**Fig. 2:**
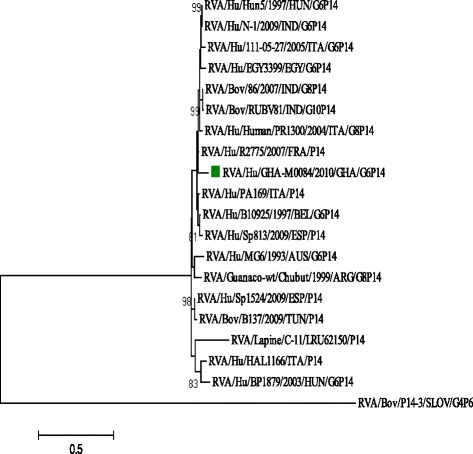
Phylogenetic tree constructed from nucleotide sequence of Ghanaian P[14] strain GHA-M0084/2010 and reference P[14] strains by the maximum likelihood method using MEGA 6.06 software and rooted with the G4P[6] rotavirus strain RVA/Bov/P14-3/SLOV/G4P6. Variation scale (nucleotide substitution per site) is indicated below the phylogenetic tree. Percentage bootstrap support is indicated by values at each node, and values ≥70 % are shown. Study strain GHA-M0084/2010 is indicated by a filled *green square*. Reference sequences used in the analysis were obtained from the GenBank database. Phylogenetic distance was measured by Kimura two-parameter model. Phylogenetic tree was supported statistically by bootstrapping with 1000 replicates

Phylogenetic analysis of the VP8* fragment of the VP4 gene of the Ghanaian strain showed it clustered together with the prototype HRV strain, PA169-88 and published HRV strains from other parts of the world (Fig. 2). It also clustered together with bovine strains, Bov/86 and RUBV81 (India) and lama guanaco strain, Chubut (Argentina) (Fig. 2). The Ghanaian VP4 gene shared the strongest nucleotide sequence identity (87.9 %) with Italian HRV strains PA169 and PR/1300 and the strongest amino acid sequence identity (89.3 %) with R2775/FRA/07 isolated in France (Table 2). However, it shared nucleotide sequence identity of ≥87.0 % ≤89.3 % with randomly selected human and animal rotavirus reference strains detected in Italy (111-05-27), France (R2775/FRA/07), India (Bov/86 & RUBV81) and in Egypt (EGY3399).

**Table 2:** Comparison of Amino acid and Nucleotide identity of Ghanaian P[14] and published P[14] rotavirus strains

Amino acid identity (%)																			
RV STRAIN	P-TYPE	MG6	Hun5	PA169	HAL1166	LRU62150	B10925	PR/1300	GHA-M0084	111-05-27	R2775	Bov/86	RUBV81	EGY3399	BP1879	Sp1524	Sp813	P14-3	B137
MG6	P[14]		68.9	68.1	60.9	50	70.4	68.9	62.6	68.9	68.9	69.1	68.4	65.4	53.7	73.1	65.9	26.4	75.1
Hun5	P[14]	88.6		86.3	68.4	52.9	85.6	87.8	73.1	92.4	89.3	87.9	88.7	90.2	55.9	70.1	82.5	24.2	69.9
PA169	P[14]	89.1	95.2		68.4	49.2	92.4	84	73.8	83.3	93.1	83.4	84.2	82.7	55.9	73.1	84	25	71.4
HAL_1166	P[14]	86	88.9	89.1		48.5	69.1	67.6	64.4	64.6	69.1	69.4	70.1	68.4	72.1	65.6	66.1	23.5	63.9
LRU62150	P[14]	80.1	80.8	79.7	79.4		50	52.2	45.9	52.9	47.7	52.5	53.2	51.4	51.4	50.3	47	20.7	50.7
B10925	P[14]	89.6	94.8	97.4	89.1	79.7		84	73.8	82.5	91.6	82.7	83.4	81.9	59.7	77.6	89.3	25	76.6
PR/1300	P[14]	88.9	95.9	94.8	88.6	80.8	94.5		73.1	86.3	87.8	84.9	85.7	84.2	56.7	69.4	78	24.2	68.4
**GHA-M0084**	P[14]	83.2	87.7	87.9	83.9	75.4	87.7	87.9		70.8	76.8	72.5	72.5	71.1	50.7	64.4	70.8	25.7	62.9
111-05-27	P[14]	88.9	97.4	94.1	87.2	80.4	93.6	95.2	87		86.3	84.9	85.7	89.4	58.2	68.6	80.3	25.7	67.6
R2775	P[14]	88.9	96.4	97.6	89.1	79.4	96.9	96.2	89.3	95.2		87.9	88.7	87.2	57.4	72.3	83.3	25	71.4
Bov/86	P[14]	88.9	95.5	94.1	89.1	80.6	93.6	94.8	87.9	94.3	95.7		99.2	85.8	59.2	71.1	75.9	24.8	70.1
RUBV81	P[14]	88.4	95.5	94.1	89.1	81.1	93.6	94.8	87.7	94.3	95.7	99.5		86.5	60	71.8	76.6	24.1	70.8
EGY3399	P[14]	88.2	96.2	94.3	89.3	80.8	93.8	94.5	87.2	95.5	95.5	95	95		59.7	67.9	79.6	25.7	66.9
BP1879	P[14]	78.7	79.9	79.9	85.3	75.2	80.8	80.4	74.7	80.8	80.6	80.8	80.8	81.1		60.7	53.7	20.7	58.9
Sp1524	P[14]	89.6	89.6	90.3	86.7	79	91.5	89.3	84.4	88.6	90.3	89.3	89.3	88.4	80.4		73.8	21.4	91.7
Sp813	P[14]	85.6	91.2	92.2	85.6	76.6	94.1	90	85.1	90.3	91.7	88.9	88.9	90.5	76.1	88.3		25.1	72.1
P14-3	P[14]	54.2	54.4	54.9	53.7	50.9	54.9	54	53.5	55.8	54.4	54.7	54.4	54.7	50.4	52.1	52.3		22.8
B137	P[14]	90.8	90	90.5	86.7	80.4	91.9	89.8	84.1	89.1	90.8	89.8	89.8	88.9	80.6	96.2	87.7	52.8	
Nucleotide identity (%)																			

## Discussion

G6 rotaviruses have been found to be an infrequent cause of human disease. Their detection in the human population was suggestive of zoonotic transmission as they have been detected almost exclusively in artiodactyls such as cattle [8, 14]. We report the detection of a G6P[14] rotavirus strain in Ghana from the stool of an infant participating in a hospital-based diarrhea surveillance study conducted in 2010 (pre-vaccine era). This strain was detected in a locality where the human population live in close proximity with their cattle, goats, sheep and pigs. This setting and proximity provide an ideal environment for dual RV infections from human and animal sources which can lead to reassortment of genes and eventual generation of novel strains.

Phylogenetic analysis of the VP7 gene of the study strain (GHA-M0084/2010) showed clustering with artiodactyl and human G6 strains in lineage III (Fig. 1). The Ghanaian strain displayed the strongest nucleotide and amino acid sequence identity (74.9–88.1 % and 80.0–86.9 % respectively) to randomly selected non-African reference strains (Table 1). It exhibited the lowest nucleotide identity (74.9 %) to African HRV strains, BA356 (Cameroon) and 48-BF (Burkina Faso) both isolated in 2010 (Table 1). The VP8* fragment of the VP4 gene of the study strain clustered together with HRV strains detected in France (2007), Italy (1988), Spain (2007) and Belgium (1997) (Fig. 2). It displayed the strongest aa (89.3 %) sequence identity to HRV strain R2775 isolated in France and nt (87.9 %) sequence identity to HRV strains PA169 and PR/1300 isolated in Italy (Table 2).

Two paediatric rotavirus vaccines (Rotarix and RotaTeq) are available and have been introduced into national immunization programmes in many countries [16, 17]. The monovalent vaccine, Rotarix, comprises a human G1P[8] rotavirus strain, whereas the pentavalent vaccine, RotaTeq, (a human-bovine reassortant vaccine) comprises human serotypes G1, G2, G3, G4, and P[8] on a bovine strain background. Both vaccines demonstrated broad protection against the most common RVA genotypes [18]. However, their efficacy is low especially on the African continent with diverse pool of strain types [19, 20]. G6P[14] strain detected in this study shares neither G- nor P-genotype with either of the two current vaccines. Though, other rotavirus proteins such as VP6 and NSP4 have been identified to play a role in the protective immunity against rotavirus infection, it is unknown whether Rotarix will provide protection against completely heterotypic G6P[14], an animal-derived human rotavirus strain.

## Conclusion

In this study, we determined the VP7 and VP4 genotypes of previously non-typeable rotavirus strain. The study reports for the first time the detection of G6P[14] rotavirus strain in a child in West Africa. Data on the impact of rotavirus vaccination globally has shown that two rotavirus vaccines, RotaTeq™ and Rotarix™, provide good homotypic and heterotypic protection. However, there are concerns about the degree of protection these vaccines provide against rare/unusual rotavirus strains such as G6P[14] that bears G/P genotype specificities not included in the vaccines. The detection of a G6P[14] strain pre-vaccine introduction in Ghana, suggests a potential compromise of vaccine effectiveness and indicates the necessity for continuous surveillance in the post vaccine era.

## Methods

### Viral RNA extraction and reverse transcription

Viral RNA was extracted from 10 % faecal suspension in phosphate buffered saline using the QIAamp viral RNA Mini kit (Qiagen/Westburg, Leusden, The Netherlands). PCR primer pairs, 9Con1/9Con2 and VP4-F/VP4-R (Additional file 1: Table S1) were used in one step RT-PCR to target the VP7 gene and the VP8* fragment of the VP4 gene to generate 903 and 663 bp respectively [21, 22]. PCR products were purified using the Exo-SAP-IT clean up kit (Affymetrix, Miles Rd Cleveland, OH, USA). Products were directly sequenced for VP7 and VP4 genes using the Bigdye terminator sequencing kit v3.1 (Perkin-Elmer Applied Biosystems, Foster City, CA, USA) on an automated Genetic analyzer ABI PRISM 3130 (Applied Biosystems).

### Nucleotide sequence and phylogenetic analysis

Chromatograms were visually inspected and edited. Genotypes were determined using the Basic Local Alignment Search Tool [BLAST] (http://blast.ncbi.nlm.nih.gov/Blast.cgi) and confirmed with RotaC v2.0 genotyping tool [23]. Edited sequences were trimmed and homology table was constructed using Bioedit v.7.0.5. The nucleotide sequences of the study strain were aligned with randomly selected cognate gene sequences available in GenBank using the ClustalW algorithm [24]. Two rotavirus reference strains; HRV serotype G2 (RVA/Human-tc/USA/DS-1/1976/G2) and bovine G4P[6] (RVA/Bov/P14-3/SLOV/G4P[6]) were included in the phylogenetic trees as out-groups for the VP7 and VP4 genes respectively (Figs. 1 and 2). Phylogenetic trees were constructed using the maximum likelihood method in MEGA v6.06 programme [25] and statistically supported by bootstrapping with 1000 replicates. Pyhlogenetic distances were calculated using the Kimura-2 parameter model.

### Nucleotide sequence accession number

The nucleotide sequences of the strain reported in this study (GHA-M0084/2010) have been deposited in the GenBank database under accession numbers KJ616414 and KJ616415 respectively.

## Additional file

Additional file 1: Table S1. List of primers used in this study. (DOCX 16 kb)

## Abbreviations

aa: Amino acid
BLAST: Basic local alignment search tool
bp: Base pair
EIA: Enzyme immunoassay
ELISA: Enzyme linked immuno-sorbent assay
G: Glycoprotein
HRVA: Human rotavirus group A
MEGA: Molecular evolutionary genetics analysis
nt: Nucleotide
P: Protease-sensitive protein
PAGE: Polyacrylamide gel electrophoresis
RNA: Ribonucleic acid
RRL: Regional reference laboratory
RT-PCR: Reverse transcription-polymerase chain reaction
RV: Rotavirus
RVA: Rotavirus group A
VP4: Viral protein 4
VP7: Viral protein 7
VP8*: Viral protein 8*
