# Supplementary material for: Detection of the first G6P[14] human rotavirus strain in an infant with diarrhoea in Ghana
Source: Virol J. 2016 Nov 10;13:183. doi: 10.1186/s12985-016-0643-y (PMC5103419; doi:10.1186/s12985-016-0643-y)
Supplement: Additional file 1: Table S1. — List of primers used in this study. (DOCX 16 kb) [file 12985_2016_643_MOESM1_ESM.docx]

| **Gene** | **Oligonucleotide Primer** | **Primer sequence (5'→3')** | **Position** | **Position** |
| --- | --- | --- | --- | --- |
| VP7 | 9Con1 | TAG CTC CTT TTA ATG TAT GG | + | 37 - 56 |
| VP7 | 9Con2 | GTA TAA AAT ACT TGC CAC CA | - | 922 - 941 |
| VP4 | VP4-F | TAT GCT CCA GTN AAT TGG | + | 132 - 149 |
| VP4 | VP4-R | TAT GCT CCA GTN AAT TGG | - | 775 - 795 |

Supplementary Table 1: List of primers used in this study
